# Reliability and Validity of Fall Efficacy Scale-International in People with Parkinson's Disease during On- and Off-Drug Phases

**DOI:** 10.1155/2019/6505232

**Published:** 2019-01-02

**Authors:** Maryam Mehdizadeh, Pablo Martinez-Martin, Seyed-Amirhasan Habibi, Seyed-Mohammad Fereshtehnejad, Amirabas Abasi, Javad Niazi Khatoon, Seyed Hassan Saneii, Ghorban Taghizadeh

**Affiliations:** ^1^Cellular and Molecular Research Center, Iran University of Medical Sciences, Tehran, Iran; ^2^Department Neuroscience, Faculty of Advance Technologies in Medicine, Iran University of Medical Sciences, Tehran, Iran; ^3^Student Research Committee, Iran University of Medical Sciences, Tehran, Iran; ^4^National Center of Epidemiology and CIBERNED, Carlos III Institute of Health, Madrid, Spain; ^5^Department of Neurology, Rasoul Akram Hospital, Iran University of Medical Science, Tehran, Iran; ^6^Division of Clinical Geriatrics, Department of Neurobiology, Care Sciences and Society (NVS), Karolinska Institutet, Stockholm, Sweden; ^7^Department of Neurology and Neurosurgery, McGill University, Montreal, QC, Canada; ^8^Division of Neurology, Faculty of Medicine, University of Ottawa, Ottawa, ON, Canada; ^9^Department of Occupational Therapy, School of Rehabilitation Sciences, Tehran University of Medical Sciences, Tehran, Iran; ^10^Department of Occupational Therapy, Faculty of Paramedicine, Mazandaran University of Medical Sciences, Sari, Iran; ^11^Department of Basic Sciences, School of Rehabilitation Sciences, Iran University of Medical Sciences, Tehran, Iran; ^12^Department of Occupational Therapy, School of Rehabilitation Sciences, Iran University of Medical Sciences, Tehran, Iran; ^13^Rehabilitation Research Center, School of Rehabilitation Sciences, Iran University of Medical Sciences, Tehran, Iran

## Abstract

**Purpose:**

Since fear of falling may be one of the main problems in people with Parkinson's disease (PD), its assessment with valid tools is necessary in both drug phases. This study was carried out to investigate the psychometric attributes of the Fall Efficacy Scale-International (FES-I) in people with PD, both in On and Off phases.

**Methods:**

One hundred twenty-four patients with PD (mean age ± standard deviation, 60.33 ± 12.59 years) were assessed with the FES-I, both in On- and Off-drug phases. Dimensionality, internal consistency, and test-retest reliability were, respectively, explored by means of factor analysis, Cronbach's alpha, and Intraclass Correlation Coefficient. Convergent validity of FES-I was established with Visual Analog Scale-Fear of Falling, Berg Balance Scale, and Functional Reach Test. Parkinson's Disease Questionnaire-39 and Unified Parkinson Disease Rating Scale-Activities of Daily Living were also applied. Discriminative validity was tested between patients with and without a history of falling.

**Results:**

Factor analysis showed two factors for On- and one factor for Off-drug phase. Internal consistency (*α* = 0.96, On phase; 0.98, Off phase) and test-retest reliability (0.94; 0.91) were satisfactory in both drug phases. There was a moderate/high correlation (*r*_S_ = |0.50–0.70|) between FES-I and Visual Analog Scale-Fear of Falling, Berg Balance Scale, and Functional Reach Test. Parkinson's Disease Questionnaire-39 and Unified Parkinson Disease Rating Scale-Activities of Daily Living were achieved in both drug phases too. The sensitivity of FES-I to discriminate Parkinson's disease with and without falls showed moderate effect size in both phases.

**Conclusion:**

This study verified that FES-I is unidimensional, reliable, and valid to measure the Fear of Falling during On- and Off-drug phases in people with PD.

## 1. Introduction

Fear of falling (FOF) in Parkinson's disease (PD) is known to be a common problem affecting about 45 to 68 percent in this population [1]. FOF is defined as a reduction in certainty in doing activities without falling (loss of self-efficacy) [2]. It may not cause disorder in daily-life by itself, but the avoidance of doing daily activities, reduction in the amount of physical activities, and increase in the risk of falling are among its serious consequences [3, 4]. In addition, the psychological impact of FOF can increase the patients' social isolation [5]. The detrimental effect of FOF on the quality of life of individuals prompts to consider this disorder an important problem requiring monitoring and treatment [6, 7].

Levodopa is the main gold standard treatment of PD and can be beneficial for almost all people with PD. However, as the disease progresses, continuous use of the drug can lead to a series of motor complications (motor fluctuations and dyskinesia). Motor fluctuations refer to oscillations between two drug phases called “On” (when the motor symptoms are controlled by the medication) and “Off” (when the motor symptoms reemerge uncontrolled by the medication) [8, 9]. Various studies have examined the effects of motor fluctuations in both drug phases on various performances such as balance and falling in patients who develop this complication. It has been shown that reducing the amount of levodopa (reaching to Off-drug phase) may cause stride time variability and increase the intensity of fear of falling and subsequent falling, which could influence results in Off state [10–12].

Several instruments have been designed to assess the fear of falling while doing daily activities [13–15]. Among these scales, Activity Balance Confidence (ABC), Fall Efficacy Scale (FES), Survey of Activities and Fear of Falling in the Elderly (SAFE), and Fall Efficacy Scale-International (FES-I) can be mentioned valid and reliable in PD during On-drug phase. The ABC scale measures the level of balance confidence while doing 16 activities without falling. ABC scale has been used widely although it contains items (such as icy pavements) that can be culturally not applicable in all populations. The FES is a well-validated and short scale that contains 10 items and measures the level of concern for falling during specific daily activities. Unfortunately, this scale does not consider social activities [16]. The SAFE scale consists of 17 items that measure the individual's avoidance of a series of activities. The main weakness of this scale is the length that may turn it impractical in some sitting [17]. One of other tools is the Fall Efficacy Scale-International (FES-I), an instrument designed by Yardley et al. in 2005. This 16-item scale has appropriate psychometric properties in elder population to measure the extent of concern caused by falling at the time of doing daily activities [18]. This scale has also been studied in people with stroke, MS, osteoporosis, and dizziness and postmenopausal women [19–22]. It was hypothesized that FES-I would be strongly correlated with scales estimating balance and would show moderate to high association with disability measures. Previous studies for dimensionality of FES-I have reported two factors (indoor and outdoor activities) in other populations [18, 22–24]. Also, with a cut-off point of 21 in FES-I total score, subjects could be divided to two groups of high and low level of fear of falling [25].

Recent studies investigated the test-retest reliability and the ability of the FES-I (and other tools for measuring FOF) for detecting PD patients with falling records during the “On” phase [26, 27]. Nevertheless, a reliable and valid instrument is required to show the extent of changes in rehabilitation and drug treatments in both drug phases [12]. While most studies in PD population have been conducted to investigate the psychometric properties of the assessment tools in “On” phase, it is unknown whether changes in the motor symptoms of patients during the “Off” phase alter the psychometric properties of the tools. Therefore, we aimed to investigate the psychometric properties (such as acceptability, dimensionality, internal consistency, test-retest reliability, and convergent and discriminative validity) of the FES-I in people with PD during both “On-” and “Off-”drug phases.

## 2. Methods

### 2.1. Subjects

One hundred twenty-four people with PD referred to movement disorder clinic in Rasoul Akram Hospital were included. Inclusion criteria were diagnosis of PD based on the UK brain bank criteria [28], fluency in Persian language, absence of evident cognitive problem (Minimal Mental Status Examination >21) [29], lack of other neurologic and orthopedic diseases (such as stroke, severe arthritis, lower limb fracture, etc.), and fixed dose of medication use (Levodopa and other dopamine agonists) until the time of retest.

All subjects signed the informed consent to take part in the project. The study protocol was approved by the ethics committee of the Student Research Committee in Iran University of Medical Sciences.

### 2.2. Assessments

The Persian version of the FES-I has been previously shown to have adequate reliability and validity in Iranian elder population [30, 31]. FES-I has 16 items that assess persons concern about falling during daily activities. Every item is scored from 0 to 4 and the maximum total score of the scale is 64, a figure representing person's fear of falling while doing activity [18].

In order to assess the convergent validity of the FES-I in both “On-” and “Off-”drug phases, we also assessed other PD disturbances relevant to falling, namely, balance, quality of life, and activities of daily living. For this purpose, the following assessments with random order were applied: Visual Analog Scale-Fear of Falling (VAS-FOF), Berg Balance Scale (BBS), Functional Reach Test (FRT), Parkinson's Disease Questionnaire-39 (PDQ-39), and Unified Parkinson Disease Rating Scale-Activities of Daily Living (UPDRS-ADL).

The VAS-FOF is a numerical scale on which elder individuals score their fear of falling in the range 0 to 10. In this scale 10 represents extreme fear of falling [32].

The BBS has proper reliability and validity in PD. This 14-item tool is designed to measure functional balance. The total score of BBS ranges from 0 to 56, and greater scores indicate better balance [33].

The FRT is used to screen persons for falling risk and to measure anteroposterior stability in PD [34]. Each subject performed 3 test trials and the average of these trials is calculated as FRT score. The persons with better functional balance receive higher score [35].

The PDQ-39 was designed to measure Parkinson's patient's quality of life. PDQ-39 consists of 8 domains (movement, daily-living activities, well-being, motivation, social support, cognition, communication, and physical discomfort). Items are scored in the range 0–4, and total scores for domains are calculated as percentage on the maximum possible score of their corresponding items, whereas the PDQ-39 summary index is the mean score of the eight domains. The lower the score, the better the quality of life [36].

The UPDRS-ADL section is commonly used to evaluate the ability of patients for doing daily-living activities. This UPDRS subscale consists of 13 items, with a total score of 0 to 52. Higher scores indicate higher disability [37].

Hoehn and Yahr (HY) scale was designed to determine the level of disease progression in people with Parkinson's. Based on this scale, the severity of disease is divided into 5 levels. At the first level, the condition is normal, and at the last level, people are wheelchair bound [38].

### 2.3. Procedures

Patients were assessed by an experienced occupational therapist through face to face interview using Persian version of the FES-I, in Off-drug phase (12 hours after taking the last dose of Levodopa and before taking the first morning dose [12]) and in On-drug phase (1 hour after taking Levodopa at first morning dose [12]). All assessments were applied during On- and Off-drug phases.

The history of falling in the last 6 months was recorded by interviewing with people with PD. Accordingly, individuals were categorized into two groups with and without history of falling. To evaluate reliability of test-retest, subjects were studied in second session (average interval, 10 days) by the same occupational therapist at the same conditions of first session.

### 2.4. Data Analysis

Normal distribution of data was tested using Shapiro–Francia test [39]. Apart descriptive statistics (mean, standard deviation, and percentage) the following statistics were used.

Acceptability was determined by floor and ceiling effects (percentages), with ≤15% values considered acceptable [40]. The range of skewness deemed acceptable was +1 to −1 [41].

Exploratory factor analysis (EFA) with varimax rotation was used to investigate the construct validity and dimensionality of FES-I. Explained variance of dimension with Eigenvalues of greater 1 was considered [42].

Internal consistency was determined through Cronbach's alpha, with a value >0.70 considered the minimum acceptable value [43]. Interitem and corrected item-total correlations were also calculated, with minimal threshold values 0.20 [44] and 0.30 [45] deemed acceptable, respectively.

Test-retest reliability of the FES-I total score was estimated by Intraclass Correlation Coefficient (ICC), two-way, and single measure, with 95% confidence interval. ICC values higher than 0.70 represent acceptable reliability [46]. FES-I precision was estimated through its Standard Error of Measurement (SEM = SD_pooled_ ∗ $\sqrt{1-\text{ICC}}$). SEM values <1/2 SD_pooled_ are considered acceptable [47].

FES-I convergent validity was explored by correlation with other measures (VAS-FOF, BBS, FRT, PDQ-39 (Domain “Mobility”), and UPDRS-ADL), using Spearman rank correlation coefficient. Correlation values higher than 0.50, between 0.35 and 0.50 and less than 0.35 were considered strong, moderate, and weak correlations, respectively [48]. It was hypothesized that FES-I would be strongly correlated with scales for estimating balance, and would show moderate to high association with disability measures.

Discriminative validity between two different groups (PD patients with and without history of falling during previous 6 months) was determined by Mann–Whitney test and Cohen's d effect size (differences of means divided by baseline pooled standard deviation). Magnitudes of effect size 0.2, 0.5, and 0.8 reflect small, moderate, and high separation power, respectively [49]. It was expected a moderate to highly significant difference of the FES-I total score between fallers and nonfallers.

Wilcoxon signed-rank test was applied to compare the scale scores between the two drug phases (significant level, *p* < 0.05) [50].

## 3. Results

The mean (±SD) age was 60.33 ± 12.59 years and disease duration was 6.66 ± 5.51 (Table 1). Thirty-four out of the 124 patients (27%) were Hoehn and Yahr (HY) stage 1; 51 patients (40.5%) in stage 2; 39 (31%) in stage 3.

Shapiro–Francia test showed nonnormal distribution of data, including the total score of the FES-I. Total FES-I scores were 27.34 and 33.38 for On- and Off-drug phases, respectively (Table 2). Fifty-three patients (42.7%) had record of falling (had history of falling during previous six months), with mean ± SD FES-I scores of 31.56 ± 10.80 and 37.81 ± 15.29 for On- and Off-drug phases, respectively, for this group.

### 3.1. Acceptability

The ceiling effect of the FES-I for both drug phases was ≥15%, but floor effect was mildly higher than this threshold (Table 2). The skewness values were in the range −1 to +1 for both On- and Off-drug phases.

### 3.2. Dimensionality

Exploratory factor analysis showed two factors for On phase and just one factor for Off phase (Kaiser–Meyer–Olkin = 0.91 and 0.95; Bartlett's test of sphericity both *p* < 0.001) (Table 3). We named the first factor as “Balance and social activities (outdoor)” which is including 8 items (7, 8, 9, 11, 13, 14, 15, and 16) and the second factor as “Basic personal activities (indoor)” which is including the remaining items (1, 2, 3, 4, 5, 6, 10, and 12).

### 3.3. Internal Consistency

Cronbach's alpha index of the FES-I was 0.96 for On- and 0.98 for Off-drug phases. Interitem correlations ranged in On state from 0.40 (Item 10 with items 14, 15, and 16) to 0.97 (Item 1 with item 7) and, in Off state, from 0.58 (Item 10 with 11) to 0.87 (Item 12 with item 16). Item homogeneity index was 0.59 for the On and 0.72 for the Off phase.

### 3.4. Reliability

In regard to the test-retest reliability for total FES-I scores, ICC values were 0.94 (CI 95% = 0.90–0.96), and 0.91 (CI 95% = 0.86–0.94) for On- and Off-drug phases, respectively. SEM calculation on these ICCs was 2.77 and 4.62, respectively, values clearly lower than 1/2 SD at first application (5.66 in On state and 7.71 in Off state).

### 3.5. Convergent Validity

This study revealed strong relationship between total FES-I score and VAS-FOF (*r* = 0.50–0.54, *p* < 0.001), tests for functional movement and its quality (BBS, FRT, PDQ-39 (Mobility) (*r* = 0.51–0.76, *p* < 0.001), and independency in daily activities (UPDRS-ADL) (*r* = 0.67, *p* < 0.001) in both On- and Off-drug phases (Table 4).

### 3.6. Discriminative Validity

The difference in FES-I scores for Fallers vs Nonfallers in On ((mean_F_ ± SD) 31.56 ± 10.80 vs 4.19 ± 10.72) and in Off, Fallers vs Nonfallers (37.81 ± 15.29 vs 30.43 ± 14.86) were statistically significant (Mann–Whithney test, *p* < 0.001 and *p* ≤ 0.005, respectively). Effect size for the FES-I in discriminating between Fallers and Nonfallers was 0.68 (On drug phase) and 0.48 (Off-drug phase).

For the complete sample of patients in the study, FES-I total score was (mean ± SD) 27.34 ± 11.32 in On and 33.38 ± 15.41 in Off state (Wilcoxon test, *p* ≤ 0.001).

## 4. Discussion

This study was carried out to assess the main clinimetric properties of FES-I for both drug phases, On and Off, in subjects with PD. Our findings revealed that FES-I has high reliability and acceptable convergent (convergent and discriminant) validity to measure fear of falling in PD during both On- and Off-drug phases.

In the FES-I (both On- and Off-drug phases), item 11 (Walking on a slippery surface) and item 14 (Walking on an uneven surface) got the highest mean score, indicating a greater degree of difficulty, according to past studies [51, 52].

Based on our test-retest results, FES-I total score was very stable in both drug phases and similar to previous report in PD populations in which is done only in On-drug phase (ICC = 0.92) [27]. Regarding the ICC obtained in the mentioned previous report, the samples were the same in size and PD duration, but in our data were included “On-” and “Off-”drug phases separately.

SEM values obtained for FES-I were ≤30% of the standard deviation at the first application. SEM value in On-drug phase is close to that obtained in Jonasson's study (2017) (SEM = 3.4) [27].

In factor analysis of FES-I total score for the drug on-phase, similar to previous studies, two dimensions have been shown (the first factor dominated by basic and instrumental activities of daily living and the second factor dominated by physically demanding outdoor activities) [18, 53]. The results factor analysis in the Off-drug phase showed unidimensionality of this scale. Possibly, patients with PD and mobility or stability problems are concerned about falling during daily activities (home or outdoor), a fact that should be considered for optimizing the management of those PD patients.

A strong correlation between total FES-I score and VAS-FOF revealed that it is reasonable to use either of these tools to measure fear of falling in both drug phases. In VAS-FOF, fear of falling is generally evaluated and can be faster. In contrast, with FES-I, fear of falling in 16 activities can be estimated with more detail by spending more time. Although both scales are used to assess the fear of falling and have a strong relationship, they also have advantages and disadvantages. FES-I score showed strong-association with other scales measuring balance (BBS, FRT, PDQ-39 (Mobility)) in both drug phases (Table 4). This finding indicates the close relationships between fear of falling and disorders of global mobility in people with Parkinson's disease that can be due to the similarities between the evaluated activities on these scales, such as up and down steps and reaching. Strong correlation between FES-I total score and scores of scales measuring independency in daily-living activities (UPDRS-ADL) showed that fear of falling is associated with restriction for performing daily activities in individuals with PD and how this factor can contribute to their functional disability and quality of life deterioration.

Findings from a previous study [27] revealed that FES-I score in On-drug phase was able to distinguish PD subjects with and without history of falling, which is aligned with our results for both drug phases. In addition, we showed that FES-I can discriminate the two drug phases, the ability with potential application for future studies to assess treatment response in PD.

Since most of the participants in our study were in early stage based on the modified Hoehn and Yahr staging, results showed a low score of the FES-I and mild floor effect of this score in both drug phases, which is in contrast to Jonasson's study (2014) in this population (in this study, PD patients were older and at higher level on modified Hoehn and Yahr.) [26]. Assessment in Off-drug phase is one of the major issues that have always been used to provide more accurate medication and evaluation clinical interventions in Parkinson's disease [10]. Examination of psychometric properties of this scale in the Off-drug phase for use in research and clinics is another strength of this study. Limitations of this study are the lack of evaluation of Levodopa dose equivalence and identification of patients with motor fluctuations. Also, using more appropriate tools for PD (e.g., the Fullerton Advanced Balance Scale or the Mini-BESTest) to analyze the FES-I convergent validity, carrying out confirmatory factor analysis (CFA) for dimensionality, and higher size of the samples are suggested for future studies addressing the properties of the FES-I in PD patients with fluctuations.

## 5. Conclusion

In conclusion, our study suggests that the FES-I is a reliable and valid scale for measuring fear of falling in individuals with PD. Also, it is an appropriate scale for clinicians and researchers to use during the On- and Off-drug phases.

## Figures and Tables

**Table 1:** Demography characteristics of people with PD (*n*=124).

Variables	Mean (±SD)
Age (year)	60.33 (±12.59)
Time since diagnosis (year)	6.66 (±5.51)
Mini mental status examination (score)	23.90 (±2.75)
*Variables*	*Frequency (%)*
Sex (male)	90 (72.58%)
Current medications	
Levodopa	101 (81.45%)
Dopamine agonists	22 (17.74%)
MAOB (monoamine oxidase enzyme-B) inhibitor	4 (3.22%)
Anticholinergics	15 (12.09%)
COMT (catechol-O-methyltransferase enzyme) inhibitor	10 (8.06%)
Affected side	
Right	65 (52.41%)
Left	59 (47.58%)
History of falling (past six months)	
No	71 (57.3%)
Yes	53 (42.7%)

**Table 2:** Characteristics statistic of Fall Efficacy Scale-International (FES-I) in patients with idiopathic Parkinson's disease (*n*=124).

Drug phase	Mean (±SD)	Range	Floor effect	Ceiling effect	Skewness	SEM	Cronbach's *α*	ICC (test-retest) (95% IC)
On	27.34 (11.32)	16–64	34.67%	1.61%	1.00	2.77	0.96	0.94 (0.90–0.96)
Off	33.38 (15.41)	16–64	21.77%	7.25%	0.64	4.62	0.98	0.91 (0.86–0.94)

SEM: standard error of measurement. 95% IC: 95% lower and upper confidence bounds for the ICC statistics are given in parentheses.

**Table 3:** Factor analysis for Fall Efficacy Scale-International (FES-I) in people with idiopathic Parkinson's disease (*n*=124).

On-drug phase			Off-drug phase	
Item of FES-I	Component (factor)		Item of FES-I	Component (factor)
	1	2		1
14	0.87		8	0.90
15	0.85		4	0.89
11	0.84		5	0.89
9	0.69		12	0.88
13	0.68		15	0.88
7	0.67		16	0.88
8	0.66		3	0.87
16	0.65		13	0.87
3		0.81	14	0.87
1		0.75	1	0.86
10		0.74	6	0.84
2		0.70	7	0.84
4		0.64	9	0.83
6		0.62	10	0.82
5		0.60	11	0.82
12		0.59	2	0.80

1: cleaning the house. 2: getting dressed or undressed. 3: preparing simple meals. 4: taking a bath or shower. 5: going to the shop. 6: getting in or out of a chair. 7: going up or down stair. 8: walking around in the neighborhood. 9: reaching for something above your head or on the ground. 10: going to answer the telephone before it stops ringing. 11: walking on a slippery surface. 12: visiting a friend or relative. 13: walking in a place with crowds. 14: walking on an uneven surface. 15: walking up or down a slope. 16: going out to a social event.

**Table 4:** Correlation between total scores of Fall Efficacy Scale-International (FES-I) with other scales in people with idiopathic Parkinson's disease (*n*=124).

Drug phase	Scales	VAS-FOF	BBS	FRT	PDQ-39 (mobility)	UPDRS-ADL
On	FES-I	0.54	−0.71	−0.51	0.65	0.59
Off		0.50	−0.70	−0.56	0.71	0.67

FES-I: Fall Efficacy Scale-International. VAS-FOF: Visual Analog Scale-Fear of Fall. BBS: Berg Balance Scale. FRT: Functional Reach Test. PDQ-39: Parkinson Disease Questionnaire-39-Domain “Mobility”. UPDRS-ADL: Unified Parkinson's disease Rating Scale-Activities of Daily Living. All coefficients, *p* < 0.001.

## Data Availability

The data used to support the findings of this study are included within the article.
